# Frequency of Meals Prepared Away from Home and Nutrient Intakes among US Adolescents (NHANES 2011–2018)

**DOI:** 10.3390/nu13114019

**Published:** 2021-11-11

**Authors:** Shauna Golper, Sayaka Nagao-Sato, Francine Overcash, Marla Reicks

**Affiliations:** Department of Food Science and Nutrition, University of Minnesota, St. Paul, MN 55108, USA; golpe002@umn.edu (S.G.); ssato@umn.edu (S.N.-S.); overc006@umn.edu (F.O.)

**Keywords:** food away from home, adolescents, nutrients, food groups

## Abstract

Frequency of consuming foods prepared away from home has been associated with lower diet quality among adults and adolescents in several earlier studies. Nutrient and food group intake and Healthy Eating Index (HEI)-2015 scores were compared among a U.S. nationally representative sample of adolescents (12–19 years, *n* = 3975) by frequency of consuming food prepared away from home ≤2 times/week (*n* = 2311) versus >2 times/week (*n* = 1664) using National Health and Nutrition Examination Survey (NHANES) data from 2011–2018. Regression analyses were used to compare intakes among adolescents by frequency of eating meals prepared away from home adjusting for covariates. Older (16–19 years) vs. younger (12–15 years) adolescents and those from homes with higher vs. lower family income to poverty ratios had greater frequency of eating meals prepared away from home. Intakes of nutrients of concern for adolescents including choline, vitamin D, potassium, magnesium, fiber, phosphorus, folate, iron, and total HEI-2015 scores and component scores for total vegetables, and greens and beans were lower among adolescents who consumed meals prepared away from home more vs. less often. However, no differences were noted among food group intakes considered good sources of nutrients of concern such as total fruit, total vegetables, whole grains, and total dairy, except for beans and peas by frequency of eating foods prepared away from home. Greater frequency of eating foods prepared away from home was associated with lower diet quality among adolescents in a nationally representative sample of U.S. adolescents.

## 1. Introduction

The U.S. Dietary Guidelines for Americans (DGA), 2020–2025 indicate that male and female adolescents are at risk of dietary inadequacy because of low intakes of nutrient-dense foods and beverages including grains, dairy, and fruits and vegetables, leading to low intakes of phosphorus, magnesium, and choline [1]. Female adolescents also have low intakes from protein subgroups resulting in underconsumption of protein, iron, folate, and vitamins B_6_ and B_12_ [1]. Other nutrients of public health concern for adolescents include calcium, vitamin D, and potassium [1]. Adolescence is a critical period for forming eating habits that contribute to growth and development in conjunction with increasing autonomy in food choices [2]. Because body mass index tracks from adolescence into adulthood [3], the basis for a healthy adult body weight is developed during adolescence. Therefore, a better understanding of factors affecting consumption of key food groups and nutrients during adolescence is needed.

Meal sources and locations are environmental factors that have been associated with adolescent diet quality and health parameters [4,5,6,7]. Dietary intake was characterized as nutritionally least favorable when a cross sectional sample of adolescents (*n* = 789) from the Baltimore-Washington DC and Seattle areas ate at locations other than home or school [4]. Similarly, less healthy food choices among a national UK sample of adolescents including chips, soft drinks, and chocolate were more likely to be consumed at other locations vs. home or vs. school/work [5]. Purchase frequency of dinner food sourced away from home was associated with greater odds of overweight/obesity, higher percent body fat and metabolic risk cluster z-scores among adolescents from two community-based obesity studies [6]. Additionally, evening meal types consumed at home including fast food, food delivered to the home, or food made from a heat-and-serve box meal were all associated with higher daily intake frequencies of less healthy food groups among a national sample of U.S. adolescents [7]. In general, meals prepared and consumed at home were likely to be healthier than meals prepared and consumed away from home.

Time spent cooking in U.S. households according to time-use surveys decreased by 20 min per day from 1985 to 2010 [8], while less time spent on home food preparation among a population-based sample of adults was associated with spending more on food away from home and using fast food restaurants more often [9]. Greater intake of fast food among a population-based cohort of tenth graders was related to lower Healthy Eating Index (HEI)-2010 scores, indicating less alignment with DGA recommendations and higher empty calorie scores [10]. The density-based (amounts per 1000 kcal) HEI is dependent on a common set of scoring standards applicable to the diets of individuals across various settings [11].

Diet quality during adolescence, based on intake of food groups that are important sources for nutrients of concern, is critical for growth and development and to establish a strong foundation for a healthy weight and overall health in adulthood. The relationship between frequency of meals prepared away from home and diet quality among adults and youth has been assessed in several studies using food consumption data from National Health and Nutrition Examination Survey (NHANES) cycles for children ages 6–18 as late as 2003–2004 and 1994–1996 for adults and children [12,13]. However, this relationship has not been explored among adolescents using more recent U.S. nationally representative NHANES data (2011–2018). Therefore, the purpose of this study was to examine associations among the frequency of meals prepared away from home, diet quality, and intake of nutrients of concern among adolescents including protein, fiber, phosphorus, magnesium, calcium, potassium, iron, choline, folate, and vitamins D, B_6_ and B_12_. The hypothesis was that adolescents who consumed meals prepared away from home more vs. less often would have lower overall diet quality and lower intakes of nutrients of concern.

## 2. Materials and Methods

### 2.1. Sample

NHANES is a U.S. nationally representative sample of non-institutionalized, civilians based on a complex multistage probability sampling design. All data are available for download from the US Centers for Disease Control and Prevention including data documentation and codebooks [14]. Data are collected in 2-year cycles.

For the current study, data from 4 cycles (2011–2018) were combined including data from 5215 adolescents 12–19 years. After excluding 1166 for non-reliable dietary recall data for day 1 or day 2 and another 74 for missing data for frequency of meals prepared away from home, data from a total of *n* = 3975 were available for analysis. The total was divided into two groups by the median frequency of meals prepared away from home, those who reported eating meals prepared away from home ≤2 meals/week (*n* = 2311) and those who reported eating meals prepared away from home >2 times per week (*n* = 1664). The frequency of adolescents who reported eating meals prepared away from home per week was 0 meals (*n* = 823), 1 meal (*n* = 694), 2 meals (*n* = 794), 3 meals (*n* = 608), 4 meals (*n* = 314), 5 meals (*n* = 259), 6 meals (*n* = 89), 7 meals (*n* = 138), and >7 meals (*n* = 256).

### 2.2. Measures

#### 2.2.1. Demographic Characteristics

Trained staff collected information via interviews administered in the home regarding demographic characteristics including adolescent age, sex, race and ethnicity, family income to poverty ratio; and the household reference (HR) person’s age, sex, education, and marital status. The HR person was defined as the household member (≥18 years) who owns or rents the residence where members reside. For adolescents 12–16 years, a proxy provided demographic data. The family income to poverty ratio was calculated by dividing family income by the U.S. Department of Health and Human Services poverty guidelines specific to the survey year. The poverty guidelines are used to determine financial eligibility for federal nutrition programs.

#### 2.2.2. Dietary Intake

The frequency of meals prepared away from home was assessed with one question in the Diet Behavior and Nutrition questionnaire as part of the Flexible Consumer Behavior Survey module developed in collaboration with the U.S. Department of Agriculture, Economic Research Service [15]: “Next I’m going to ask you about meals. By meals, I mean breakfast, lunch, and dinner. During the past 7 days, how many meals did you get that were prepared away from home in places such as restaurants, fast food places, food stands, grocery stores, or from vending machines?” Participants were asked not to include meals provided as part of school lunch or breakfast. Participants responded with a range of values from 0 to 21. Data from those who had more than 21 meals per week, refused to answer, or did not know were not included in the analyses. Participants who were 12–15 years answered this question in the mobile examination center (MEC), while those 16–19 answered in the home interview.

Day 1 24-h dietary recall interviews were conducted by trained staff in the MEC using a 5-step Automated Multiple Pass Method (AMPM) [16]. The interviews were conducted in a private room in the MEC using special measuring tools to help identify the appropriate portion sizes of the food eaten. After the initial day 1 dietary interview, a second day 2 dietary interview was conducted to obtain a more complete picture of dietary intake of adolescents in the U.S. The second dietary interview was done over the phone. Participants were given measuring cups, spoons, a ruler, and food model booklet to use for recalling the appropriate food measurements on the phone. Reliable dietary recalls were characterized as those where the participant had completed the first 4 steps in the AMPM and had identified food and beverages consumed for each reported eating occasion. Adolescents were interviewed directly regarding dietary intake, comprising 99% of the respondents for day 1 and 98% for day 2 recall interviews.

After the dietary recall information was obtained from the two interviews, the amounts of macronutrients and micronutrients consumed were calculated based on the Food and Nutrient Database for Dietary Studies [17]. The nutrients examined in the current study were those identified as nutrients of concern for adolescents by the DGA, 2020–2025 including protein, fiber, vitamins B_6_, B_12_, and D, choline, folate, calcium, phosphorus, potassium, magnesium, and iron [1].

Foods and beverages consumed were converted into 37 USDA food pattern components as part of the Food Patterns Equivalents Database (FPED) [18]. Specific food patterns are measured as food group servings (cup equivalents of fruit, vegetables, beans and peas, and dairy; ounce equivalents of grains and protein foods; teaspoon equivalents of added sugars; and gram equivalents of solid fats). Food group intakes evaluated in the current study were those considered good sources of nutrients lacking among adolescent diets including total fruits, total vegetables, whole grains, total protein foods, total dairy, and those that contribute to lower diet quality (solid fats and added sugars) [1].

The total Healthy Eating Index (HEI)-2015 score and adequacy and moderation component scores were calculated using the National Institutes of Health, National Cancer Institute Statistical Analysis System (SAS) code [19] to measure diet quality based on alignment with key recommendations in the 2015–2020 Dietary Guidelines for Americans [11]. Nine adequacy component scores included total fruit, whole fruit, total vegetables, greens and beans, whole grains, dairy, total protein foods, seafood and plant proteins, and fatty acids. Four moderation component scores included refined grains, sodium, added sugars, and saturated fats. For both adequacy and moderation component scores, higher scores indicated greater alignment.

#### 2.2.3. Data Analysis

SAS Survey statistical software was used to analyze data (version 9.4, SAS Analytic Software, Cary NC, USA). The SAS Surveyfreq procedure was used to describe the categorical variables including demographic data from adolescents and HR persons’ characteristics. The Rao-Scott chi square test was used to determine associations among categorical variables by frequency of meals prepared away from home per week (≤2 meals/week vs. >2 meals/week). SAS Surveymeans was used to determine the mean dietary intakes and standard errors of nutrients, food groups, and HEI-2015 scores by frequency of meals prepared away from home. Linear regression analyses were performed using SAS Surveyreg with nutrients, food groups, and HEI-2015 scores as dependent variables and frequency of meals prepared away from home as the independent variable. Regression models were adjusted for covariates including adolescent age, sex, race and ethnicity, and energy intake (except for HEI scores, which are calculated based on amounts per 1000 kcal), HR person’s age, and family income to poverty ratio. HR person’s education was not included as a covariate because it was highly correlated with family income to poverty ratio. The statistical significance level was set at 0.05 with a Bonferroni Holm adjustment for multiple comparisons. All analyses included appropriate cluster, stratum, and weight statements to account for the complex probability sampling design.

## 3. Results

### 3.1. Demographic Characteristics

About half of all adolescents were 12–15 years of age (51%) (Table 1). Those who had meals prepared away from home >2 times per week were likely to be older (16–19 years) compared to younger adolescents (12–15 years) (*p* < 0.0001). More than half of non-Hispanic White adolescents (57%) consumed meals prepared away from home >2 times per week compared to lower percentages of Mexican American and other Hispanic (19%), non-Hispanic Black (14%), non-Hispanic Asian (4%), and other races, including multi-racial (5%) adolescents (*p* = 0.003). Slightly less than half (47%) of all adolescents lived in homes with a ratio of family income to poverty (PIR) <1.3 which was considered low income [20]. Adolescents in families who had a PIR ratio ≥3.5 (high income) were more likely to have meals prepared away from home than adolescents in families with a PIR ratio <1.3 (low income) and ≥1.3 to ≤3.49 (middle income) (*p* = 0.0002). Adolescents in homes with older household reference (HR) persons (40–59 years) were more likely to have more meals prepared away from home than adolescents in homes with younger HR persons (20–39 years) (*p* < 0.0001). Adolescents in homes with more educated HR persons were more likely to have more frequent meals away from home than those in homes with less educated HR persons (*p* = 0.002). No differences were observed in frequency of meals prepared away from home by adolescent sex, HR person’s sex, or marital status.

### 3.2. Dietary Intakes

Intakes of choline (*p* < 0.0001), vitamin D (*p* < 0.0001), potassium (*p* < 0.0001), magnesium (*p* = <0.0001), fiber (*p* = 0.002), phosphorus (*p* = 0.002), folate (*p* = 0.009), and iron (*p* = 0.007) were lower among adolescents who consumed meals prepared away from home more vs. less often (Table 2). No differences were observed in intakes of protein (*p* = 0.018), vitamins B_6_ (*p* = 0.016) or B_12_ (*p* = 0.052), or calcium *(p* = 0.053) by frequency of consuming meals prepared away from home.

Consumption of total fruit (*p* = 0.011) and total vegetables (*p* = 0.013) was not lower among those eating meals prepared away from home >2 times per week compared to those eating meals prepared away from home less often after Bonferroni–Holm correction for multiple comparisons (Table 3). Intake of beans and peas computed as vegetables was significantly lower (*p* = 0.001) for those eating meals prepared away from home more vs. less often. Intakes of other food groups including whole grains (*p* = 0.063), total dairy (*p* = 0.063), total protein foods (0.251), solid fats (*p* = 0.883), and added sugars (*p* = 0.078) were not significantly different by frequency of eating meals prepared away from home.

The total HEI score (*p* < 0.0001) and component scores for total vegetables (*p* = 0.003) and greens and beans (*p* = 0.002) were lower (indicating less alignment with DGA) among those eating meals prepared away from home >2 times per week compared to those eating meals prepared away from home less often (Table 4). Component scores for whole fruit (*p* = 0.010) were marginally significantly lower for adolescents eating meals prepared away from home more vs. less often. Other components scores were not different by frequency of eating meals prepared away from home.

## 4. Discussion

This study addressed the associations between the frequency of meals prepared away from home, diet quality, and intakes of nutrients of concern [1] among adolescents using NHANES data (2011–2018). Frequency of meals prepared away from home was associated with several demographic characteristics including adolescent and HR person’s age, family income to poverty ratio, and adolescent race and ethnicity. The study hypothesis was supported by lower overall diet quality and lower intakes of nutrients of concern among adolescents who consumed meals prepared away from home more vs. less often.

About two-thirds (66.6%) of all U.S. adolescents were estimated to have poor diet quality based on NHANES 2015–2016 [21] consistent with the current study where all adolescent participants had a mean total HEI-2015 score of 47.8. Because eating meals prepared away from home more vs. less often was associated with poorer diet quality among adolescents, home cooking and consuming meals prepared at home may enhance diet quality and should be promoted in public health education efforts. For example, Supplemental Food and Nutrition Assistance Program education (SNAP-Ed) routinely provides cooking education and information regarding strategies to stretch the food dollar by preparing healthy foods at home [22]. Participation in the Food Stamp program (now called SNAP) among U.S. adults was associated with fewer meals prepared away from home based on NHANES 2003–2004 [23]. Similarly, those adolescents in the current study in families with the lowest compared to highest family income to poverty ratio were less likely to eat meals prepared away from home, which may partially offset the disparities in diet quality among youth by income level. According to Liu et al. [21], nearly two-thirds (64.5%) of youth (2–19 years) with household income <1.3 times the poverty level had poor diet quality compared to 47.2% of those with household income ≥3.0 times the poverty level. However, other factors in addition to whether meals are prepared away from home or at home are also likely to influence disparities in diet quality by income level, such as the availability of neighborhood supermarkets vs. convenience stores, and time spent working outside the home.

In the current study, older (16–19 years) vs. younger (12–15 years) adolescents were more likely to consume meals prepared away from home, such as from restaurants, fast food places, food stands, grocery stores, or from vending machines. When foods are consumed away from home, parental control of availability may be less influential than when foods are consumed at home, especially among older adolescents who have more autonomy in food selection and greater opportunities to consume foods away from home. A systematic review and meta-analysis showed that parental control of availability (whether foods are available in the home) was an important predictor of healthy and unhealthy food consumption by both children and adolescents [24]. In the current study, greater frequency of meals prepared away from home among adolescents 16–19 years compared to those 12–15 years indicated that diet quality may be less influenced by parents and therefore, more compromised among older vs. younger adolescents.

Nutritional requirements increase during adolescence to support growth and development including increases in longitudinal height, body weight, peak bone mass, muscle mass, blood volume, and organ size [25]. In the current study, intakes of several nutrients important for these functions were lower for those with higher frequency of meals prepared away from home including vitamin D, potassium, phosphorus, iron, and folate. Vitamin D and phosphorus along with calcium play a crucial role in bone accretion and optimal peak bone mass in adolescence [26]. Among adolescents, potassium intake is linked to blood pressure management [27]. Iron requirements are increased to support increased muscle mass and higher hemoglobin levels [25]. In addition, deficiencies of some nutrients can limit availability for critical functions. For example, in winter months in the Northeastern U.S., 75% of urban children (9–14 years) were found to be vitamin D deficient [28] and 13% of U.S. adolescent girls were iron deficient based on having abnormal values for 2 of 3 ferritin indicators according to NHANES 2003–2006 data [29]. Therefore, the clinical significance of the findings for adolescents regarding differences in nutrient intakes by frequency of meals prepared away from home may vary by stage of development and vitamin and mineral status.

Only a small percentage (2.0%) of U.S. adolescents reported meeting DGA vegetable recommendations [1] according to the U.S. Youth Risk Behavior Surveillance System, 2017 [30]. Similarly, in the current study, the HEI-2015 component scores for total vegetables, and greens and beans were low (2.45/5, and 1.25/5, respectively) among all adolescents in the current study and lower for those eating meals prepared away from home more vs. less often. Consuming evening meals cooked from scratch was associated with increased daily intake of fruit and vegetables among a national sample of adolescents [7]. In addition, cooking interventions based on mobile apps and gardening led to greater incorporation of vegetables into meals among low-income parents and youth [31] and increased vegetable consumption among older children [32], respectively. While vegetables are generally consumed at low levels by adolescents, they are good sources of nutrients such as fiber, potassium, and folate, which were consumed at lower levels when meals prepared away from home were consumed more vs. less often in the current study. Therefore, community-based interventions based on effective strategies to improve vegetable intake among adolescents should be encouraged.

This study includes several limitations. Adolescents were grouped using median frequency of meals prepared away from home, which did not consider other environmental contextual factors including the type of location and people present at the eating occasions. The 24-h recall data may include biases, based on accuracy of memory, social desirability factors, and day-to-day variability [33]. In addition, non-reliable dietary recall data for days 1 and 2 were excluded from a large number of adolescents (*n* = 1166). The data used in the current study were collected from adolescents before the COVID-19 pandemic (2011–2018). COVID-19-related changes in the way food is acquired by consumers, increases in food insecurity and disruptions in supply chains may have affected the influence of consuming meals prepared away from home on diet quality for different population subgroups. Therefore, the findings from the current study may not be as applicable to current food acquisition/preparation patterns of U.S. adolescents.

NHANES data (2015–2016) for adolescents showed that the percentages of nutrients from food and beverages consumed away from home (any location other than home) including vitamin D, potassium, magnesium, choline, phosphorus, iron, and folate were all consumed at levels between 26% and 41% per day [34]. Therefore, future research directions could include development and testing of interventions to improve adolescent selection of foods prepared away from home to increase intake of those foods considered good sources of nutrients of concern. Cooking education and gardening interventions could also be developed and tested to increase frequency of home cooking and therefore, intake of vegetables, and greens and beans among adolescents.

## Figures and Tables

**Table 1:** Demographic characteristics of U.S. adolescents by frequency of meals prepared away from home.

Characteristic	All *n* (% ^3^) *n* = 3975	≤2 Meals/Week ^1^ *n* (% ^3^) *n* = 2311	>2 Meals/Week *n* (% ^3^) *n* = 1664	*p*-Value ^2^
Adolescent age group				<0.0001
12–15 years	2023 (50.6)	1373 (60.2)	650 (39.0)	
16–19 years	1952 (49.4)	938 (39.8)	1014 (61.0)	
Adolescent sex				0.289
Male	1971 (51.0)	1162 (52.2)	809 (49.4)	
Female	2004 (49.0)	1149 (47.8)	855 (50.6)	
Adolescent race/ethnicity				0.003
Mexican American/Other Hispanic	1212 (30.4)	743 (24.4)	469 (19.3)	
Non-Hispanic White	1039 (26.1)	580 (50.3)	459 (57.3)	
Non-Hispanic Black	1016 (25.6)	583 (15.2)	433 (13.8)	
Non-Hispanic Asian	463 (11.6)	271 (5.0)	192 (4.2)	
Other race-including multi-racial	245 (6.2)	134 (5.1)	111 (5.4)	
Ratio of family income to poverty				0.0002
<1.3 low	1856 (46.7)	1120 (38.0)	736 (32.4)	
≥1.3 to ≤3.49 middle	1338 (33.7)	786 (37.5)	552 (33.7)	
≥3.5 high	781 (19.6)	405 (24.5)	376 (33.8)	
HR ^4^ person’s sex				0.550
Male	1790 (45.0)	1061 (48.2)	729 (49.6)	
Female	2185 (55.0)	1250 (51.7)	935 (50.4)	
HR person’s age group				<0.0001
<20 years	177 (4.5)	56 (2.2)	121 (6.7)	
20–39 years	1141 (28.7)	724 (29.0)	417 (20.4)	
40–59 years	2350 (59.1)	1362 (61.7)	988 (66.1)	
60+ years	307 (7.7)	169 (7.1)	138 (6.7)	
HR person’s marital status (*n* = 205 missing)				0.050
Married/living with partner	2596 (68.9)	1586 (75.8)	1010 (71.8)	
Widowed/divorced/separated	821 (21.8)	450 (17.9)	371 (21.0)	
Never married	353 (9.4)	199 (6.2)	154 (7.2)	
HR person’s education (*n* = 149 missing)				0.002
Less than high school	511 (13.4)	336 (12.8)	175 (8.2)	
High school graduate/GED ^4^ or some college/AA ^4^ degree	2687 (70.2)	1545 (69.5)	1142 (69.4)	
College graduate or above	628 (16.4)	345 (17.8)	283 (22.4)	

^1^ Number of meals (breakfast, lunch, dinner) per week prepared away from home in places such as restaurants, fast food places, food stands, grocery stores, or from vending machines (does not include meals provided as part of the school lunch or school breakfast); ^2^ *p*-value based on Rao-Scott chi square tests; ^3^ weighted column percentages; ^4^ HR = household reference, GED = general education development, AA = associate’s of arts.

**Table 2:** Nutrient intakes among U.S. adolescents by frequency of meals prepared away from home.

Nutrients	All Mean (SE ^1^) *n* = 3975	≤2 Meals/Week ^2^ LS ^1^ Means (SE) *n* = 2311	>2 Meals/Week LS Means (SE) *n* = 1664	*p*-Value ^3^
Vitamin D (D_2_ + D_3_) (μg)	4.8 (0.1)	5.4 (0.2)	4.4 (0.2)	**<0.0001**
Potassium (mg)	2199.9 (23.4)	2251.1 (24.1)	2112.0 (26.2)	**<0.0001**
Magnesium (mg)	245.4 (3.2)	248.6 (4.7)	229.1 (3.3)	**<0.0001**
Choline (mg)	269.6 (3.8)	276.6 (4.9)	254.0 (4.5)	**<0.0001**
Phosphorus (mg)	1311.8 (17.0)	1319.2 (17.2)	1265.7 (15.8)	**0.002**
Fiber (gm)	14.6 (0.2)	14.8 (0.3)	13.7 (0.2)	**0.002**
Iron (mg)	14.8 (0.2)	15.4 (0.4)	14.0 (0.3)	**0.007**
Folate (μg)	387.6 (6.0)	400.3 (8.7)	368.1 (7.8)	**0.009**
Vitamin B_6_ (mg)	1.97 (0.04)	2.03 (0.17)	1.88 (0.18)	0.016
Protein (gm)	74.9 (0.9)	75.3 (0.9)	72.9 (1.1)	0.018
Vitamin B_12_ (μg)	5.1 (0.1)	5.3 (0.2)	5.0 (0.2)	0.052
Calcium (mg)	995.7 (15.4)	1004.4 (20.5)	963.8 (21.5)	0.053

^1^ SE = standard error, LS mean = least squares mean; ^2^ number of meals (breakfast, lunch, dinner) per week prepared away from home in places such as restaurants, fast food places, food stands, grocery stores, or from vending machines (does not include meals provided as part of the school lunch or school breakfast); ^3^ *p*-value based on linear regression models adjusted for adolescent age, sex, race/ethnicity, and energy intake, family income to poverty ratio, and household reference person’s age. Bonferroni–Holm correction was applied for multiple comparisons (bolded *p*-value indicates statistical significance).

**Table 3:** Food group intakes among U.S. adolescents by frequency of meals prepared away from home.

Food Groups	All Mean (SE ^1^) *n* = 3975	≤2 Meals/Week ^2^ LS ^1^ Means (SE) *n* = 2311	>2 Meals/Week LS Means (SE) *n* = 1664	*p*-Value ^3^
Total fruit ^4^, cup eq	0.86 (0.03)	0.89 (0.09)	0.79 (0.10)	0.011
Total vegetables ^4^, cup eq	1.02 (0.02)	1.06 (0.07)	0.98 (0.08)	0.013
Beans and peas ^4^, cup eq	0.08 (0.01)	0.09 (0.01)	0.06 (0.02)	**0.001**
Whole grains ^4^, oz eq	0.85 (0.03)	0.82 (0.08)	0.76 (0.08)	0.063
Total dairy ^4^, cup eq	1.90 (0.04)	1.98 (0.13)	1.87 (0.16)	0.063
Total protein foods ^4^, oz eq	5.0 (0.1)	5.0 (0.1)	4.8 (0.2)	0.251
Solid fats ^4^, g	34.8 (0.5)	34.2 (0.5)	34.5 (0.6)	0.883
Added sugars ^4^, tsp eq	17.87(0.3)	17.6 (0.5)	18.5 (0.4)	0.078

^1^ SE = standard error, LS mean = least squares mean; ^2^ number of meals (breakfast, lunch, dinner) per week prepared away from home in places such as restaurants, fast food places, food stands, grocery stores, or from vending machines (does not include meals provided as part of the school lunch or school breakfast); ^3^ *p*-value based on linear regression models adjusted for adolescent age, sex, race/ethnicity and energy intake, family income to poverty ratio, and household reference person’s age. Bonferroni Holm correction was applied for multiple comparisons (bolded *p*-value indicates statistical significance); ^4^ Total fruit (intact fruits (whole or cut) and fruit juice), total vegetables (total dark green, red and orange, starchy, and other vegetables; excludes legumes), beans and peas (legumes computed as vegetables), whole grains (defined as whole and contains the entire grain kernel), total dairy (total milk, yogurt, cheese, and whey), total protein foods (total meat, poultry, organ meat, cured meat, seafood, eggs, soy, and nuts and seeds; excludes legumes), solid fats (naturally present in meat, poultry, eggs, and dairy (lard, tallow, and butter); fully or partially hydrogenated oils; shortening; palm oil; palm kernel oil; coconut oils; fats naturally present in coconut meat and cocoa butter; and 50% of the fat present in stick and tub margarines and margarine spreads), added sugars (caloric sweeteners such as syrups and sugars).

**Table 4:** HEI-2015 scores among U.S. adolescents by frequency of meals prepared away from home.

Food Groups	Maximum Score	All Mean (SE ^1^) *n* = 3975	≤2 Meals/Week ^2^ LS ^1^ Means (SE) *n* = 2311	>2 Meals/Week LS Means (SE) *n* = 1664	*p*-Value ^3^
Total HEI score	100	47.8 (0.3)	49.0 (0.5)	46.1 (0.5)	**<0.0001**
**Adequacy**					
Total fruits	5	2.23 (0.07)	2.33 (0.10)	2.08 (0.08)	0.024
Whole fruits	5	2.17 (0.07)	2.28 (0.11)	1.94 (0.07)	0.010
Total vegetables	5	2.45 (0.04)	2.60 (0.05)	2.35 (0.07)	**0.003**
Greens and beans	5	1.25 (0.05)	1.40 (0.09)	1.04 (0.07)	**0.002**
Whole grains	10	2.81 (0.09)	2.77 (0.12)	2.49 (0.14)	0.144
Dairy	10	6.51 (0.09)	6.75 (0.15)	6.45 (0.16)	0.152
Total protein foods	5	4.10 (0.04)	4.17 (0.04)	4.00 (0.07)	0.054
Seafood and plant protein	5	2.06 (0.05)	2.13 (0.08)	1.87 (0.09)	0.019
Fatty acids	10	4.10 (0.09)	4.12 (0.14)	4.25 (0.17)	0.564
**Moderation**					
Refined grains	10	4.76 (0.08)	4.98 (0.11)	4.50 (0.15)	0.025
Sodium	10	3.94 (0.09)	3.82 (0.13)	3.79 (0.13)	0.850
Added sugars	10	6.05 (0.08)	6.06 (0.14)	5.83 (0.13)	0.275
Saturated fats	10	5.36 (0.08)	5.57 (0.13)	5.52 (0.13)	0.821

^1^ SE = standard error, LS mean = least squares mean; ^2^ number of meals (breakfast, lunch, dinner) per week prepared away from home in places such as restaurants, fast food places, food stands, grocery stores, or from vending machines (does not include meals provided as part of the school lunch or school breakfast); ^3^ *p*-value based on linear regression models adjusted for adolescent age, sex and race/ethnicity, family income to poverty ratio, and household reference person’s age. Bonferroni Holm correction was applied for multiple comparisons (bolded *p*-value indicates statistical significance).

## Data Availability

Center for Disease Control and Prevention. National Health and Nutrition Examination Survey 2011–2018. NHANES Questionnaires, Datasets, and Related Documentation. Available online: https://wwwn.cdc.gov/nchs/nhanes/ (assessed on 11 October 2021).
